# American Medical Association

**Published:** 1859-04

**Authors:** S. M. Bemiss

**Affiliations:** Sec’y. Am. Med. Asso.


### AMERICAN MEDICAL ASSOCIATION.

The twelfth annual meeting of this association will be held in Louisville, Kentucky, on Tuesday, May 3d, 1859. The secretaries of all societies, and other bodies entitled to representation in the association, are requested to forward to the secretary, S. M. Bemiss, at Louisville, correct lists of their delegations as soon as they are appointed.

The convention of teachers, invoked by a resolution of the national association, for the purpose of a general conference upon the best means of elevating the standard of medical education in this country, will meet in the same city on Monday, the 2d of May.

Medical journals throughout the United States are requested to insert the above.

S. M. BEMISS, Sec'y. Am. Med. Asso.

